# Telomere shortening in leukocyte subpopulations in depression

**DOI:** 10.1186/1471-244X-14-192

**Published:** 2014-07-05

**Authors:** Alexander Karabatsiakis, Iris-Tatjana Kolassa, Stephan Kolassa, K Lenhard Rudolph, Detlef E Dietrich

**Affiliations:** 1 Clinical & Biological Psychology, Institute of Psychology and Education, University of Ulm, Ulm, Germany; 2 SAP Switzerland, Tägerwilen, Switzerland; 3 Leibniz Institute for Age Research, Fritz Lipman Institute, Jena, Germany; 4 Burghof-Klinik, Rinteln, Germany; 5 Clinic for Mental Health, Hannover Medical School, Hannover, Germany

**Keywords:** Depression, Telomere shortening, Aging, Stress, Immunosenescence

## Abstract

**Background:**

Telomere shortening is a normal age-related process. However, premature shortening of telomeres in leukocytes – as has been reported in depression – may increase the risk for age-related diseases. While previous studies investigated telomere length in peripheral blood mononuclear cells (PBMCs) as a whole, this study investigated specific changes in the clonal composition of white blood cells of the adaptive immune system (CD4+ helper and CD8+ cytotoxic T lymphocytes, and CD20+ B lymphocytes).

**Methods:**

Forty-four females with a history of unipolar depression were investigated and compared to fifty age-matched female controls. Telomere lengths were compared between three groups: 1) individuals with a history of depression but currently no clinically relevant depressive symptoms, 2) individuals with a history of depression with relevant symptoms of depression, and 3) healthy age-matched controls. Telomere length was assessed using quantitative fluorescence *in situ* hybridization (qFISH).

**Results:**

Both groups with a history of unipolar depression (with and without current depressive symptoms) showed significantly shorter telomeres in all three lymphocyte subpopulations. The effect was stronger in CD8+ and CD20+ cells than in CD4+ cells. Individuals with a history of depression and with (without) current symptoms exhibited a CD8+ telomere length shortening corresponding to an age differential of 27.9 (25.3) years.

**Conclusions:**

A history of depression is associated with shortened telomeres in the main effector populations of the adaptive immune system. Shorter telomeres seem to persist in individuals with lifetime depression independently of the severity of depressive symptoms. CD8+ cytotoxic T cells and CD20+ B cells seem to be particularly affected in depression. The total number of depressive episodes did not influence telomere length in the investigated adaptive immune cell populations.

## Background

Depression may be associated with an accelerated cellular aging of the immune system, contributing to a higher morbidity and mortality due to an earlier onset of age-related diseases such as cardiovascular diseases [1], and cancer [2]. Telomere length (TL) represents a biological marker of aging [3]. Telomeres shorten in healthy aging in CD4+ T helper, CD8+ cytotoxic T cells, and antibody-producing B cells at a yearly rate of 19-35 base pairs [4]. Telomere shortening increases the risk for age-related diseases such as cardiovascular diseases [1] and cancer [5]. Animal studies showed that chronic stress accelerates telomere shortening [6]. In human studies, shorter telomeres were found in pessimistic women [7], after psychological stress, including a history of early maltreatment [8], severe social deprivation [9], and in patients with mood disorders [10,11]. Epel et al. [12] showed telomere shortening in individuals with greater environmental exposure to stress (mothers with a chronically ill child), which was not due to changes in the subcomposition of peripheral blood mononuclear cells (PBMCs). In sum, several studies point towards an acceleration of cellular aging due to psychological distress. One contributing factor how psychological stress might be translated to accelerated aging is via increased rates of reactive oxygen (ROS) and reactive nitrogen species (RNS). Both lead to unspecific damage of intracellular compartments including cell membranes, organelles, DNA and also telomeres [13,14]. Cells counteract these processes with a limited potency of antioxidant protection [15]. Another contributing factor of shorter telomeres in depression might be the reported increase in the total number of immune cells in depression [16] as the higher cell division rates would lead to increasing cell numbers implying an accelerated loss of telomeres. Finally, due to the higher risk of suffering from physical diseases, depressive individuals might possibly have a higher wear and tear (due to clonal expansion) of adaptive immune cells, contributing to telomere shortening [10].

To know which specific cells out of the different cell types that PBMCs consist of are most affected by telomere loss, probably reflecting premature immunological aging, would be of high scientific relevance. Only two studies so far differentiated telomere length attrition in different cell types in the context of healthy [17] and pathological aging [18]. Results suggest that the (CD8+ cytotoxic) T cells are most affected by premature aging.

This study investigated telomere length in cells of the adaptive immune system, namely CD4+ helper T cells, CD8+ cytotoxic T cells and CD20+ B cells in individuals with a history of unipolar depression vs. controls. Three groups were distinguished: 1) individuals with a history of unipolar depression and no clinically relevant symptoms of depression, 2) individuals with a history of unipolar depression with relevant symptoms of depression, and 3) age-matched healthy controls. We hypothesized that individuals with a history of depression (both with and without relevant symptoms of depression) show significantly shorter telomere length in all three lymphocyte subpopulations in comparison to the control group.

## Methods

### Participants

Forty-four individuals with at least one episode of unipolar depression (ICD-10 diagnosis: F32.0-2, F32.4; F33.0-2, F33.4), as determined by standard clinical evaluation and documented in clinical records, and 50 control subjects who reported no current or past psychiatric disorder participated in the study. Both groups were matched for age (range: 39-66 years) and education. Middle to age-progressed individuals were recruited as depression-related telomere shortening was assumed to have an increasing effect with age. To exclude gender-specific effects on telomere shortening all participants were females recruited by newspaper announcement and public advertisements. Following an initial telephone screening in which basic exclusion criteria were applied (age, history of stroke, head injuries or trauma, epilepsy or any similar neurologic disease, loss of consciousness for more than 10 minutes), qualifying participants were invited to donate 50 ml of peripheral blood by venous puncture. Depressive symptoms were assessed by the *Beck Depression Inventory* (BDI) [19]. A sum score below 14 in the BDI has been suggested as an indicator of remission [20]. Based on the BDI score, subjects with lifetime depression were divided into two subgroups: 1) Individuals with irrelevant severity of depressive symptoms (BDI ≤ 13, [IS]) and 2) lifetime depressed with relevant depressive symptoms (BDI ≥ 14, [RS]). Participants were further interviewed about their medical history. Participants who reported a lifetime or concurrent general medical, neurological or psychiatric disorders (e.g. dementia, Parkinson’s disease, non-comorbid anxiety disorders including post-traumatic stress disorder, Borderline personality disorder or different types of phobia) were excluded from the study. Inclusion criteria for the group of lifetime depressed was at least one unipolar depressive episode and a minimum period of six months since the last hospitalization in a psychiatric clinic according to the release letter they provided. The age at first depressive episode and the total number of episodes were assessed using the provided clinical documentation of hospitalization. In addition, participants were interviewed about their clinical history. All participants gave written informed consent and received a compensation of 50 €. The study was approved by the Ethics Committee of the Hannover Medical School.

### Isolation of leukocyte subfractions and purity control

Blood samples were taken into EDTA buffered blood collection tubes (Sarstedt, Germany) and processed with standard procedures by Ficoll-Paque (GE Healthcare, USA) dense-gradient centrifugation to isolate buffy coat. Blood was collected between 10 am and 1 pm. Participants were allowed to eat and drink in the morning. Leukocytic subpopulations (CD4+ and CD8+ T- and CD20+ B-cells) were isolated by MACS separation using appropriate magnetically labeled antibody beads (Miltenyi Biotech, USA) following the manufacturer’s protocol. The purity of the isolated cell fractions was evaluated using appropriate antibodies (Miltenyi Biotech, USA) at distinct intervals by cytometry performed on randomly selected isolates. As telomeres were measured in isolated subpopulations of white blood cells (WBCs), possible stress-induced alterations in the composition of WBCs can be excluded and cannot influence the telomere length results observed in this study. Cells were washed three times with standard phosphate buffered saline followed by fixation using a methanol/glacial acetic acid solution (3:1). Fixated cells were stored at -80°C prior to fluorescence microscopy analysis. TL was assessed using 10^5^ cells per sample dropped onto superfrost slides (Menzel-Gläser, Germany).

### Telomere length analysis

Quantitative fluorescence *in situ* hybridization (qFISH) was performed using peptide nucleic acid telomere oligonucleotides (Applied Biosystems, USA) labeled to the fluorescent dye Cy-3. The resulting telomere fluorescence intensity (TFI) signal correlates with TL. Co-staining with DAPI (Vectashield, USA) was performed to ensure the integrity of the cells by excluding false-positive perinuclear signals. Digital images were captured with a thousandfold optical magnification by a digital camera. The camera was mounted on a fluorescence microscope equipped with the appropriate Cy3-specific filters and connected to the Leica image control software (Leica microscope DM5000B, Leica, Germany). The optimal exposure time was determined prior to the study by trial and error using corresponding leukocytic subcells of one 30 years old healthy male donor that were also used as an internal control and reference (set to TFI = 100%). By this means image capturing was performed under standardized technical conditions (exposure time: 250 milliseconds; microscope sensitivity threshold level = 2). TFI was assessed in monochrome images relating telomere length to pixel brightness. The image acquisition software TFL-TeloV2 [21] was used to estimate TFI. Telomere length was calculated as the percentage difference between the mean TL of the corresponding cell population from the internal control and the individual mean TL of each set of cells from one subject. Telomere length was estimated in 75 cells of the individual leukocyte subset per subject. Samples with less than 75 assessed cells were excluded from analysis (see Table 1).

**Table 1:** Results of the clinical assessment and telomere length (TL) comparison between control subjects (CG), lifetime depressed with irrelevant symptoms (IS) and with relevant symptoms of depression (RS)

	**Group**	**N**	**Mean**	**SD**	**Overall test**	**CG vs. IS**	**IS vs. RS**
Age (years)	CG	50	51.1	8	χ^2^_2_ = 2.10,		
	IS	24	53.1	7.2	*p* = .35		
	RS	20	53.8	7.6			
Education (years)	CG	50	15.1	2.4	χ^2^_2_ = 3.05,		
	IS	24	14.1	2.1	*p* = .22		
	RS	20	14.2	2.9			
Age at first depressive episode (years)	CG	50	-				
	IS	24	37.2	12.6			*t*_36.8_ = 1.33,
	RS	20	31.5	15.4			*p* = .19
Total depressive episodes	CG	50	-	-			
	IS	24	2.4	1.0			*W* = 176.5,
	RS	20	3.2	2.1			*p* = .27
BDI	CG	50	3.2	2.8	χ^2^_2_ = 53.7,	*W* = 253,	*W* = 5.5,
	IS	24	7.5	4.8	*p* < .0001	*p* = .0003	*p* < .0001
	RS	20	21.7	7.4			
SSRI/ SNRI intake	CG	0 of 50					
	IS	10 of 24					*χ*^2^_2_ = .33,
	RS	9 of 20					*p* = .56
L-Thyroxine intake	CG	3 of 50			χ^2^_2_ = 1.26,		
	IS	1 of 24			*p* = .53		
	RS	0 of 20					
CD4+ TL (%)	CG	48	67.77	16.05	See text for detailed models and results		
	IS	24	55.06	15.73			
	RS	20	53.09	17.88			
CD8+ TL (%)	CG	45	68.7	13.69	See text for detailed models and results		
	IS	24	51.1	11.98			
	RS	17	48.76	13.07			
CD20+ TL (%)	CG	44	68.83	16.83	See text for detailed models and results		
	IS	23	47.44	9.44			
	RS	16	50	13.69			
Hypertension	CG	1 of 50					
	IS	1 of 24					
	RS	2 of 20					

TL of the reference sample (30 years old healthy male subject) was set to 100%. TL of CD4+, CD8+, and CD20+ cells given in % related to the reference. BDI, Beck Depression Inventory; SSRI, selective serotonine reuptake inhibitors; SNRI, serotonine and norepinephrine reuptake inhibitors; L-Thyroxine, treatment for hypothyreosis.

### Statistics

Statistical analyses were performed using R 3.0.2 [22]. Linear model residuals were tested for normality [23] and equality of variance [24]. We used *Kruskal-Wallis* or *Wilcoxon* tests when preconditions for ANOVA were not fulfilled.

## Results

### Demographical data and clinical assessment

Groups did not differ with respect to age or education (see Table 1). Furthermore, IS and RS groups did not differ significantly in mean age at the first depressive episode or in total number of depressive episodes. As would be expected, the three groups differed significantly in the BDI: controls showed lower BDI compared to the IS group, which in turn showed lower BDI than the RS group. IS and RS groups did not differ on SSRI intake. The three groups did not differ in L-thyroxine intake for the treatment of hypothyreosis.

### Statistical analysis of telomere length

For each cell type, we calculated three different models with the dependent variable telomere length: 1) with a factor groups alone, 2) with a factor group and a covariate age with homogeneous slopes between groups, and 3) with an interaction between group and age. The model with a homogeneous influence of age yielded the smallest *Akaike information criterion* (AIC) [25] for CD8+ and CD20+ cells, whereas the model with an interaction between groups and age yielded a very slightly lower AIC for CD4+ (-64.7 without, -65.1 with the interaction). We report on the model with homogeneous age influence for all cell types below, as results do not change appreciably with the other candidate models. The *Shapiro-Wilk* test indicated that residuals from all three models were not normally distributed for the CD4+ and the CD20+, but a *Kruskal-Wallis* nonparametric test yielded similar results for group effects. In addition, we verified the results by substituting the BDI for group membership in our models while controlling for age, although this modification led to clearly larger AICs.

**Figure 1:**
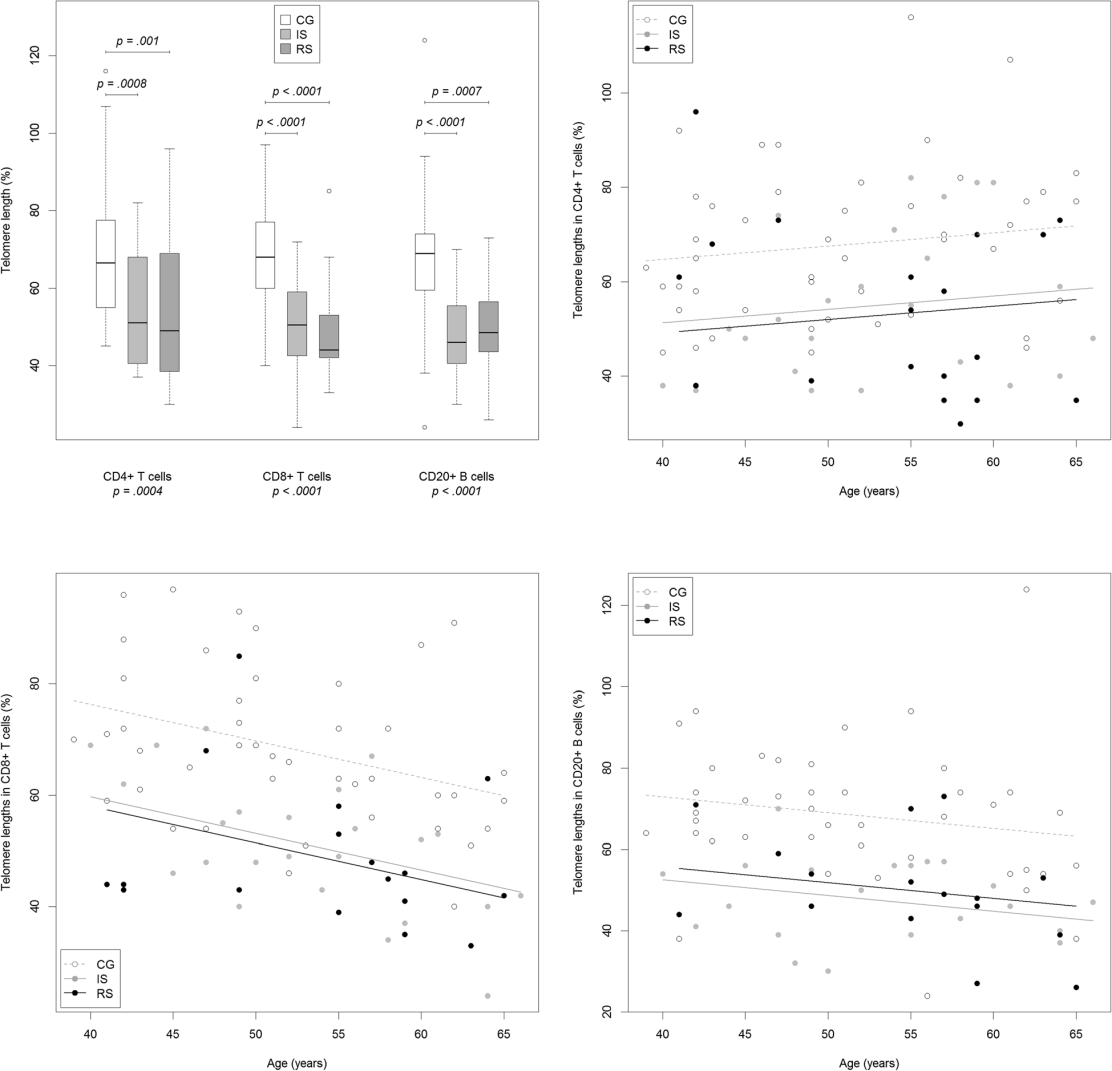
**Between-group comparison of telomere length and age-related changes in adaptive immune cells.** Top left: boxplot of telomere lengths by group and cell type. P values for groups as calculated in ANOVAs, with age as a covariate. Outliers were included into the analysis. Top right, bottom left and bottom right: observed telomere lengths per cell type in CD4+, CD8+, and CB20+ lymphocytes (as percentages of telomere lengths of a reference sample, see text) together with group-wise model fits against age (lines). Model fits span ages observed within each group. CG: control group; IS: lifetime depressed with irrelevant symptoms of depression (BDI ≤ 13); RS: lifetime depressed with relevant symptoms of depression (BDI ≥ 14).

For CD4+ cells, there was a significant effect of group, *F*(2,87) = 8.54, *p* = .0004, *η*^*2*^ = .16, *η*^*2*^_*p*_ = .16, but not of age, *F*(1,87) = .31, *p* = .58, *η*^*2*^ = .003, *η*^*2*^_*p*_ = .004. Post hoc tests revealed significant differences between CG and IS, *F*(1,68) = 12.28, *p* = .0008, as well as between CG and RS, *F*(1,64) = 11.24, *p* = .001, but not between IS and RS, *F*(1,41) = .15, *p* = .70. For CD8+ cells, there were significant effects of group, *F*(2,81) = 20.89, *p* < .0001, *η*^*2*^ = .29, *η*^*2*^_*p*_ = .34, and of age, *F*(1,81) = 20.63, *p* < .0001, *η*^*2*^ = .14, *η*^*2*^_*p*_ = .20. Post hoc tests revealed significant differences between CG and IS, *F*(1,65) = *28.*86, *p < .*0001, as well as between CG and RS, *F(1,58) =* 24.32, *p < .*0001, but not between IS and RS, *F*(1,38) *= .*21 , *p = .*65. To compare the TL reduction observed in the IS and RS to the one associated with aging, the coefficient estimate of the linear model for TL corresponding to each group (-.167 for IS, -.184 for RS), giving the group effect, was divided by the coefficient estimate for age (-.00657), giving the TL reduction per year of age (see Figure 1). To assess the variability of this statistic, it was bootstrapped 10,000 times. The TL reduction in IS (RS) participants corresponded to the reduction in control participants -.167/-.00657 = 25.3 (-.184/-.00657 = 27.9) years older (slight differences due to floating point arithmetic and rounding), bootstrapped 95% confidence interval 13.9-58.6 (12.3-66.9). For CD20+ cells, there were significant effects of group, *F*(2,78) = 17.82, *p* < .0001, *η*^*2*^ = .30, *η*^*2*^_*p*_ = .31 and age, *F*(1,78) = 7.03, *p* = .01, *η*^*2*^ = .06, *η*^*2*^_*p*_ = .08. Post hoc tests revealed significant differences between CG and IS, *F*(1,63) = 28.64, *p <* .0001, as well as between CG and RS, *F*(1,56) = 12.79, *p =* .0007, but not between IS and RS, *F*(1,36) *=* .79, *p =* .38. As expected, the BDI had significant effects on telomere lengths in all three cell types: *F*(1,85) = 12.04, *p* = .0008, *η*^*2*^ = .12, *η*^*2*^_*p*_ = .12 for CD4+, *F*(1,79) = 21.53, *p* < .0001, *η*^*2*^ = .18, *η*^*2*^_*p*_ = .21 for CD8+ and *F*(1,76) = 7.50, *p* = .008, *η*^*2*^ = .09, *η*^*2*^_*p*_ = .09 for CD20+. The total number of past depressive episodes (CD4+: *p* = .95, CD8+: .27, CD20+ 0.83), antidepressant medication (SSRI/SNRI; CD4+: *p* = .76, CD8+: *p* = .92, CD20+: *p* = .41) and L-thyroxine intake (CD4+: *p* = .26, CD8+: *p* = .57, CD20+: *p* = .63) did not change the pattern of results or became significant when included in models.

## Discussion

Individuals with a history of unipolar depression (whether with or without clinically relevant symptoms of depression) exhibited shorter telomeres in the three analyzed leukocyte subsets (CD4+ T helper cells, CD8+ cytotoxic T cells, and CD20+ B cells). As we investigated telomere length in subpopulations, the telomere length results cannot be influenced by stress-induced alterations in the composition of WBCs, which has been reported in stress- and trauma-related research [26,35]. However, the common investigation of PBMCs as a whole in contrast to subpopulations might explain some of the inconsistencies in telomere-related findings in depression. Effect sizes (*η*^*2*^/*η*^*2*^_*p*_) indicate that depression has a stronger effect in CD8+ and CD20+ than in CD4+ cells. Our results support the assumption of a systemic effect of depression on telomere length in the adaptive immune system [27]. The IS group, although they did not show clinically relevant depressive symptoms any more, and in particular the RS group were still significantly more depressed than the control group. As both groups did not differ significantly in telomere length, depression-associated TL shortening did not significantly recover since the last depressive episode in comparison to the controls – assuming no differences in pre-depression TL. Our results support the findings of Verhoeven and colleagues [28] who also investigated telomere length in depression and found no differences between current and remitted depressed individuals. However, we can of course not rule out that telomere shortening might be reversible. Future studies should longitudinally follow up after remission of depression.

### Limitations

We did not assess body mass index, nutrition, and life style factors (tobacco use, alcohol consumption), which might also influence telomere length. We also did not assess the menstrual/hormonal status of participants, hypertension or cardiovascular diseases, all of which may influence mood and contribute to the etiology of depression [29]. As only women were investigated in this study, the findings cannot be extrapolated to men. Gender-specific differences in telomere length dynamics should be investigated in the future. Participants were asked about current medication intake but not about lifetime medication. Furthermore, we did not assess the effect of nutritional supplements on telomere length dynamics. This would be an interesting aspect for further investigations. In addition, we assessed the age at depression onset and the total number of depressive episodes only based on the provided clinical documentation. Although our findings that the number of depressive episodes did not influence telomere length is in line with the work of Hartmann and colleagues [30] who reported that telomere shortening in depression is independent from illness duration, we cannot rule out that the assessed information was rather unreliable and thus might have distorted a true effect of disease duration as well as number of depressive episodes on telomere length. Similarly, we did not assess in the completely remitted depressed group the time interval since the last depressive episode. This should be investigated in future studies. Finally, both clinical groups still, even those without clinically relevant symptoms, showed higher depressive symptoms than the control group. Residual depressive symptomatology is often observed in individuals with a history of unipolar depression [31-34]. Thus, it is possible, although unlikely, that in both clinical groups telomere length could recover, if they reached BDI scores comparable to those of controls. Therefore longitudinal studies with depressed individuals over the course of treatment should investigate telomere dynamics in healthy and psychopathological aging of the (adaptive) immune system.

### Future studies

In future studies it would be interesting to further subdivide cytotoxic T cells in naïve and memory T cells. Naïve T cells decrease with healthy aging [35]. Indeed, a specific reduction in naïve cytotoxic T cells has been observed in patients with posttraumatic stress disorder [36] suggesting an accelerated aging of the adaptive immune as a consequence of (traumatic) stress. Furthermore, future studies should also assess telomerase activity, the enzyme that elongates telomeric structures, in cells of the adaptive immune system, as we know by now that telomerase is active in stimulated lymphocytes [17,4]. Further, Choi and colleagues have shown that cortisol impairs *telomerase* activity in human T lymphocytes [37]. Finally, telomere shortening might be observed in various psychiatric disorders (e.g. anxiety disorders [38], post-traumatic stress disorder [8]) and should therefore be investigated in various stress-related psychiatric diagnoses in the future. Moreover, future studies should also investigate telomere shortening not only in immune cells but also in cells of different organs. Indeed, telomeric length between different organs correlates in an individual [39,40]. Therefore, it is possible that individuals with a history of depression would also show shorter telomeres in other organs and systems.

## Conclusions

Individuals with a history of depression show shortened telomeres in the main effector populations of the adaptive immune system and shorter telomeres seem to persist independently of the severity of depressive symptoms. CD8+ cytotoxic T cells and CD20+ B cells seem to be particularly affected in depression. The total number of clinically documented depressive episodes did not influence the telomere length in depressed individuals.

## Competing interests

All authors declare that they have no competing interests.

## Authors' contributions

AK coordinated the study, performed the data collection, and conducted all biological analyses. AK and ITK wrote and revised the manuscript. DED and KLR designed the study. DED provided funding. KLR provided scientific and technical support for telomere length determination. SK performed the statistical analyses together with AK and ITK. All authors read and approved the final manuscript.

## Pre-publication history

The pre-publication history for this paper can be accessed here:

http://www.biomedcentral.com/1471-244X/14/192/prepub
